# May Diet and Dietary Supplements Improve the Wellness of Multiple Sclerosis Patients? A Molecular Approach

**DOI:** 10.4061/2010/249842

**Published:** 2011-02-24

**Authors:** Paolo Riccio, Rocco Rossano, Grazia Maria Liuzzi

**Affiliations:** ^1^Dipartimento di Biologia D.B.A.F., Università degli Studi della Basilicata, 85100 Potenza, Italy; ^2^Istituto Nazionale di Biostrutture e Biosistemi (INBB), 00100 Roma, Italy; ^3^Dipartimento di Biochimica e Biologia Molecolare “Ernesto Quagliariello”, 70126, Bari, Italy

## Abstract

Multiple sclerosis is a complex and multifactorial neurological disease, and nutrition is one of the environmental factors possibly involved in its pathogenesis. At present, the role of nutrition is unclear, and MS therapy is not associated to a particular diet. MS clinical trials based on specific diets or dietary supplements are very few and in some cases controversial. To understand how diet can influence the course of MS and improve the wellness of MS patients, it is necessary to identify the dietary molecules, their targets and the molecular mechanisms involved in the control of the disease. The aim of this paper is to provide a molecular basis for the nutritional intervention in MS by evaluating at molecular level the effect of dietary molecules on the inflammatory and autoimmune processes involved in the disease.

## 1. The Etiopathogenesis of Multiple Sclerosis: A Disease with Significant Neurological Disability in Young Adults

Multiple sclerosis (MS) is a chronic, demyelinating disease of the Central Nervous System (CNS) in young adults [1]. It is generally accepted that MS is an inflammatory and autoimmune disease, characterized by blood brain barrier (BBB) disruption, perivascular inflammation, axonal and oligodendrocyte injury, and breakdown of the myelinsheath [2]. In particular, autoreactive CD4^+^ T cells, directed towards the myelin sheath, pass through the BBB and together with macrophages and microglial cells degrade the myelin sheath [3]. Mounting evidences suggest the involvement in MS pathogenesis of other adaptive immune cells, such as Th17 cells and B lymphocytes, and innate immune cells, such as dendritic cells, natural killer T cells, and resident microglia [4]. Another pattern of both myelin and oligodendrocyte damage is mediated by antibodies or complement activation [5]. MS is also considered a neurodegenerative disease, with axonal damage occurring very early in the course of the disease [6].

MS is a complex, heterogenous and multifactorial disease with unknown etiology. Dysregulation of the immune response, genetic predisposition and environmental factors (infectious and/or nutritional) are possible causative agents, but none of these factors alone can explain its origin [7, 8]. Environmental factors act at a prodromal stage for the disease, long before that MS becomes clinically evident, and for this reason their causal pathways are difficult to determine [9]. 

It has been suggested that the persistence of particular viruses, in particular the Epstein-Barr virus [10–12], the human endogenous retroviral family W (HERV-W) elements [13, 14], other microbial agents, or toxins may represent a causative condition for MS in genetically suited individuals. However, there are no data yet concerning the direct involvement of a specific virus in MS, and the relevance of viral infection could be ascribed more to the age and persistence of infection rather than to a particular virus [9]. 

On the contrary, the uneven geographical distribution of the disease and the influence of migration in young age on the course of MS, strongly suggest a relevant role of noninfectious environmental factors such the nutritional habits and duration of exposure to the sunlight. 

According to the 2008 MS Atlas of the World Health Organization (WHO) and the Multiple Sclerosis International Federation (MSIF)—downloaded from http://www.who.int/mental_health/neurology/en/—MS is indeed prevalent in the more developed Western countries furthest from Equator [15, 16]. If the genetic background is not the discriminating element, susceptibility to MS might be then determined by the high-fat/high-carbohydrate and hypercaloric “Western” diets, typical of countries with high incomes, rather than by microbial infections. On the other hand, latitude and reduced sun exposure might influence the availability of vitamin D.

The aim of this short review is to furnish a molecular basis for a nutritional intervention in MS. This is an important task because the relevance of nutrition in MS has not been established yet, and studies on the relationship between diet and MS are very few [17–21]. At present, MS therapy is not associated to a particular diet, but the majority (about 70%) of MS patients try complementary and alternative medicine (CAM) treatments, often without informing their physician [22–24]. 

On the other hand, the suggestion of a particular diet might be not sufficient. To demonstrate the influence of nutrition on MS, it is necessary to assess at the molecular level the safety and the effectiveness of nutritional interventions, including the administration of specific dietary supplements. In other words, we need to identify the dietary molecules able to influence the course of the disease, their targets in the cell, and the molecular mechanisms involved.

## 2. The Impact of Dietary Molecules on Cell Metabolism

**Figure 1:**
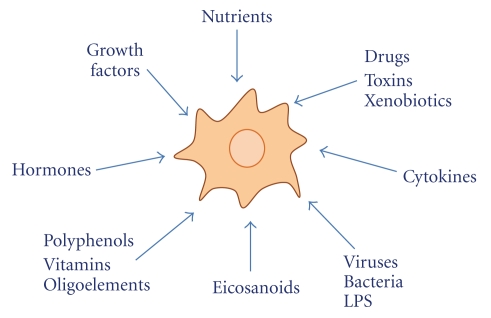
Cells are integrated networks constantly responding to environmental stimuli. The ability to recognize changes in environmental conditions and to adapt themselves to those changes, is essential for the viability of cells, and changes in quality and quantity of dietary molecules are the most frequent in the course of the day.

The question arises as to whether and how dietary molecules exert their influence on cell activity. First of all, cells certainly have the necessary machinery to adapt themselves to changes in their environment, and changes in content and type of dietary molecules are the most common over time (Figure 1).

Fundamentally, the molecular mechanisms by which dietary molecules can influence the performance of our organism in health and disease are those primarily involved in the control of metabolism and energy homeostasis. There are three main types of control of cell activity that can be exerted by food molecules: (1) direct binding to key regulatory enzymes (allosteric modulation); (2) posttranslational modifications of enzymes or proteins in general (proteolytic cleavage, or enzyme phosphorylation, glycosylation, methylation, acetylation …); and (3) modification of transcriptional regulation affecting enzyme/protein expression [25]. 

The influence of dietary molecules on cell activity is certainly very complex and involves a number of targets and multiple and intricate signaling pathways in the cell. 

**Figure 2:**
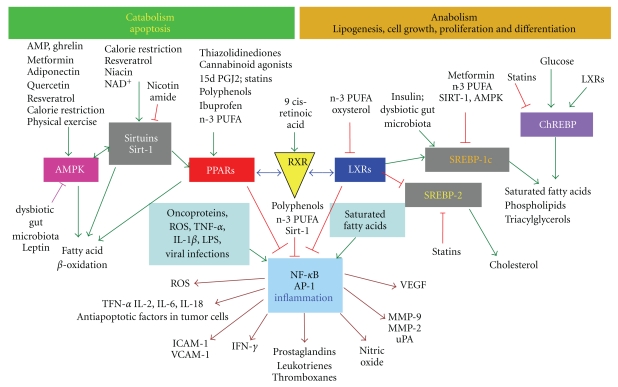
Schematic representation of biological responses to nutrients and cellular outcomes. Influence of some natural dietary compounds and some common drugs on cell transcriptional activity in metabolism and inflammation. PPAR: peroxisome proliferator activated receptor; LXR: liver X receptor; RXR: retinoid X-receptor; NF-*κ*B: nuclear transcription factor-*κ*B; SREBP: steroid regulatory element-binding protein; ChREBP: carbohydrate responsive element-binding protein; Sirtuins: SIRT-1/2 deacetylating enzyme; AMPK: AMP Protein Kinase; MMP: metalloproteinase; VEGF: vascular endothelial growth factor; TNF: tumor necrosis factor; ROS: reactive oxygen species; ICAM*-*1: intercellular adhesion molecule: VCAM*-*1: vascular adhesion molecule; n-3 PUFA, omega-3 polyunsaturated fatty acids.

Targets and metabolic pathways are depicted in the simplified scheme of Figure 2. On the left, low intake of nutrients, calorie restriction or fasting, and physical exercise upregulate oxidative metabolism and metabolic rate to match energy demand. On the right, high intake of energy-rich nutrients upregulates the anabolic pathways, lipogenesis, cell growth, cell differentiation and proliferation, as well as proimatory events. 

**Figure 3:**
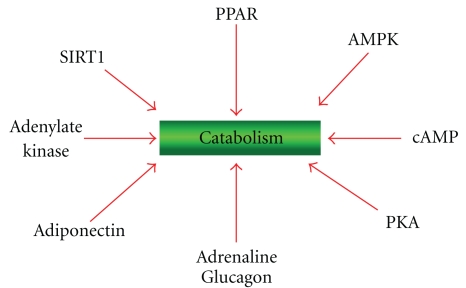
Enzymes, hormones, and transcription factors involved in catabolism. PKA: protein Kinase A, cAMP dependent. AMPK: 5′ AMP-activated protein kinase.

**Figure 4:**
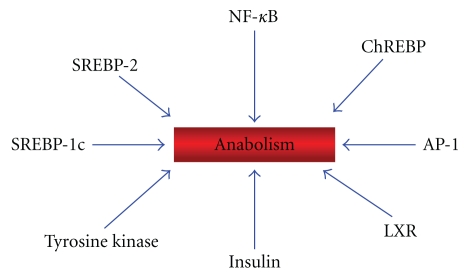
Enzymes, hormones and transcription factors involved in anabolism.

The switch between catabolism and anabolism is determined by specific sensors that allow the cells to adapt themselves to changes of nutrients in their surrounding environment shifting their metabolism either to the catabolic (Figure 3) or to the anabolic pathways (Figure 4). These sensors are ligand-dependent, multidomain nuclear receptors, transcription factors, and enzymes. Upon activation by binding of lipids, cholesterol derivatives, glucose, vitamins, hormones or antioxidants, transcription factors bind to DNA and regulate gene expression and nutrient metabolism. 

Among the most important metabolic nuclear receptors there are the *peroxisome proliferator-activated receptors* (PPARs) [http://ppar.cas.psu.edu] and the *liver X receptors* (LXRs). Both compete with *retinoid X receptor* isotypes *α*, *β*, *γ* (RXRs) to form active heterodimers and are involved, but in different way, in the regulation of fatty acid metabolism (Figure 2). 

PPAR isotypes *α*, *β*/*δ*, *γ* function as lipid sensors and respond to the increase of fatty acids (and in particular of oxidized fatty acids) by upregulating the transcription of genes involved in catabolism, in particular fatty acid beta-oxidation in mitochondria and peroxisomes [26, 27]. 

Other important metabolic sensors involved in catabolism are the enzymes Sirtuins, a family of seven NAD^+^-dependent deacetylase enzymes [28] and the AMP kinases (AMPK) [29, 30]. The cross talk between PPAR, AMPK and sirtuins (SIRT), induces mitochondrial biogenesis and fatty acid oxidation [28, 31]. 

Figure 4, and the right side of the panel in Figure 2 show the transcription factors and enzymes involved in lipogenesis. LXRs are lipogenic transcription factors activated by the cholesterol derivatives oxysterols and glucose [32]. LXRs facilitate the synthesis of fatty acids and triacylglycerols through the activation of the *steroid regulatory element-binding protein 1c* (SREBP-1c), while inhibiting SREBP-2 and thereby the synthesis of cholesterol. LXRs also activate the *carbohydrate response element binding protein* (ChREBP), which regulate metabolic gene expression to convert excess carbohydrate into triglyceride rather than glycogen. 

In conclusion, the switch between catabolism and anabolism is determined by the choice of the RXR binding partner, either PPAR (catabolism) or LXR (anabolism). 

## 3. The Impact of Dietary Molecules on Inflammation

The link between the above transcription factors and the nutrients explain how cells regulate energy homeostatis and respond to changes in nutritional status, but represents also the molecular key to understand how nutrients can influence the course of chronic inflammatory diseases, as they can also downregulate the proimatory transcription factors. 

The key inflammatory transcription factors involved in the inflammatory responses are shown at the center of Figure 2. They are the Activator Protein-1 (AP-1) and the Nuclear transcription Factor k of activated B cells (NF-*κ*B). In particular, NF-*κ*B is activated by ROS, endotoxins, inflammatory stimuli, X-rays, carcinogens and viruses, dissociates from its inhibitory proteins I*κ*B, and translocates to the nucleus, where it induces the expression of more than 200 genes related to cell proliferation, metastasis, and inflammation.

In MS, AP-1 and NF-*κ*B are activated [33] and specific enzymes and their products involved in inflammation are overexpressed: matrix metalloproteinase-9 (MMP-9, gelatinase B), plasminogen activators, phospholipase A_2_ (PLA_2_), cyclooxigenase-2 (COX-2) and prostaglandins, lipoxygenases (LOX) and leukotrienes, Th1 cytokines, IL-1 and TNF, adhesion molecules, reactive oxygen species (ROS), as well as nitric oxide synthase (NOS) and nitric oxide. As a consequence, inflammation, oxidative stress, and angiogenesis are increased or sustained. Oligodendrocytes, neurons and the myelin sheath are injured. Microglial cells sustain the neuroinflammatory processes.

At the molecular level, the links between metabolism and inflammation are given by the nuclear receptors PPAR and LXR. Besides their role as regulators of energy homeostasis and metabolic diseases, PPAR and LXR isotypes also have an important role in inflammatory pathways and immunological regulation. PPARs exert antiinflammatory effects by inhibiting NF-*κ*B and AP-1 [34] and are able to integrate metabolic and inflammatory signaling by inhibiting inflammatory gene expression [35].

## 4. Nutrients to Avoid in MS

### 4.1. Animal Fat

In principle, MS patients should avoid the intake of food containing molecules that are potentially harmful over time and should prefer healthy food containing those molecules that can improve their well-being by lowering, for example, the extent of inflammation.

**Figure 5:**
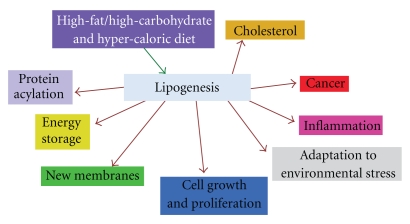
Metabolic patterns and diseases correlated with lipogenesis.

Among the dietary factors that have been considered most frequently for their deleterious influence on MS are saturated fatty acids of animal origin (found in whole milk, butter, cheese, meat, sausages …). In 1950 Swank suggested that the consumption of saturated animal fat is directly related to the frequency of MS [36]. In 2003, Swank and Goodwin reported that restriction of saturated fat induces remission of the disease and produces beneficial effects in MS patients [37]. These effects have been ascribed to the fact that saturated fat forms aggregates that may be not capable of entering the smallest capillaries [38]. Obstructions of the capillaries may contribute to MS and other diseases. In addition saturated fats decrease membrane fluidity, lead to the synthesis of cholesterol, activate the CD14/TLR4 receptor complex and favor the formation of TNF-*α*. As shown in Figure 5, hypercaloric diets rich in saturated lipids and lipogenesis favor several human diseases.

It is becoming clear now that the influence of fat on cells is more direct: is controlled at transcriptional level [25], and influences gene expression, cell metabolism, and cell growth, and differentiation [39]. 

### 4.2. Cow Milk, MFGM Proteins, and Molecular Mimicry in MS

MS is believed to be an autoimmune disease. Environmental (either microbial or dietary) factors can be associated with autoimmunity and myelin breakdown by molecular mimicry, that is, the amino acid homology between the autoantigen and microbial or dietary peptides [40]. According to this hypothesis, molecular mimicry may disrupt immunological self-tolerance to CNS myelin antigens in genetically susceptible individuals.

The best example of potential molecular mimicry between myelin autoantigens and dietary proteins is given by cow's milk. The hypothesis of a link between milk consumption and MS has been taken into consideration since the mid of 1970s [41, 42]. Later, epidemiological studies gave support to this hypothesis [43].

The milk proteins that could be detrimental in MS are the proteins of the milk fat globule membrane (MFGM proteins) [44]. The MFGM proteins account for only 1-2% of the total protein fraction, and for this reason they are not relevant for their nutritional value [45].

The protein which is most frequently suspected of the association with MS is butyrophilin (BTN), the most representative MFGM protein. BTN belongs to the Ig superfamily and is very similar to MOG, the myelin oligodendrocyte glycoprotein, one of the candidate autoantigen in MS [26]. BTN inhibits the MOG-induced experimental autoimmune encephalomyelitis (EAE), but also induces inflammatory responses in the CNS, when injected alone into animals, and stimulates *in vitro* MOG-specific T cell responses [46]. Antibody cross-reactivity between MOG and BTN has been observed in MS [47, 48]. Antibodies against BTN and other MFGM proteins have been detected also in two other diseases: autism [49] and coronary heart disease (CHD) [50].

The MFGM proteins might have detrimental effects on health also because they are associated to milk saturated fat, another possibly deleterious dietary component. On these grounds, we have introduced the concept that the consumption of the MFGM proteins by MS patients should be discouraged [44]. Different is the opinion of Spitsberg (2005) who reviewed the intake of MFGM proteins and milk fat and recommended them for their potential nutraceutical value [51]. However, reports on the relationship between human health and cow milk do not substantiate such an assertion, at present.

## 5. Dietary Molecules Promoting the Wellness of MS Patients

### 5.1. Bioactive Natural Compounds for the Complementary Treatment of MS

Specific bioactive dietary molecules could counteract the effects of microbial agents and downregulate the expression of inflammatory molecules. There are indeed several natural compounds which are able to interfere with cell signaling and counteract oxidative stress, angiogenesis and the production of inflammatory molecules which are associated with MS. Among them, the most important compounds are dietary molecules such as polyphenols and carotenoids from vegetables, polyunsaturated fatty acids (PUFA) from fish, vitamin D, and elements such as selenium and zinc. 

#### 5.1.1. Antioxidant Dietary Molecules: Polyphenols and Carotenoids

**Figure 6:**
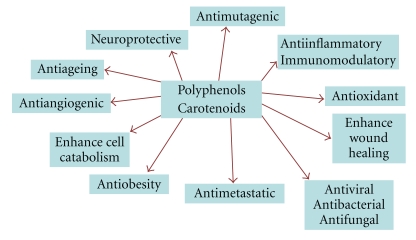
General biological activities of polyphenols & carotenoids.

Polyphenols and carotenoids are bioactive molecules found in vegetables, fruits, wine and other fruit-based beverages, spices, and herbs. These dietary molecules are well known for their antioxidant activity, but recent findings indicate that they may have additional properties independent of their roles as antioxidants and radical scavengers (Figure 6). 

The rationale for the use of natural antioxidants in MS is based on the finding that oxidative stress and in particular the generation of reactive oxygen species (ROS), is one of the most important components involved in inflammation and neuronal damage [52, 53]. On these grounds, the administration of appropriate doses of antioxidants, such as the dietary polyphenols and carotenoids, could be very useful to restore the right balance between ROS generation and their destruction.

**Figure 7:**
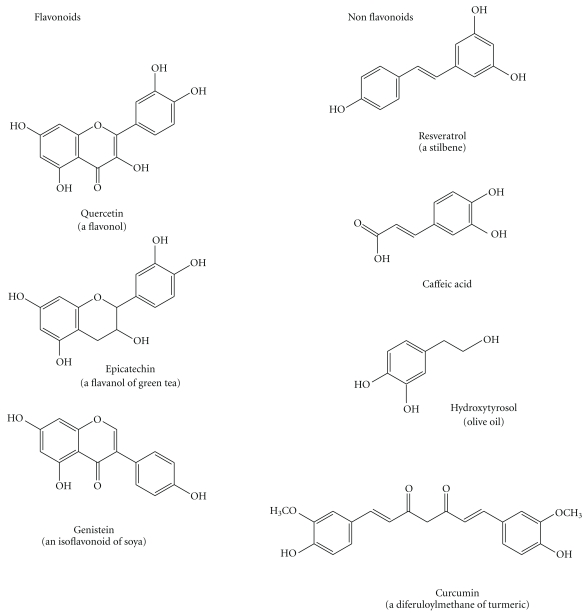
Structure of the most important polyphenols.

Polyphenols include flavonoids and nonflavonoids molecules (Figure 7). Flavonoids, the most abundant polyphenols in plants, are more than 8000 different compounds which have in common the structure of diphenylpropanes (C6-C3-C6) with two benzene rings separated by a linear three-carbon chain forming an oxygenated heterocycle. Flavonoids can be divided into various classes: flavanols (catechin, epigallocatechin), flavones (luteolin, apigenin), flavanones (naringenin, hesperidin), flavonols (quercetin, myricetin), isoflavones (daidzein, genistein) and anthocyanidins (cyanidin, malvidin) [54, 55]. 

Nonflavonoids are hydroxycinnamic or phenolic acids (caffeic acid, ferulic acid, including curcumin, a diferuloylmethane), lignans (secoisolariciresinol), stilbenes (resveratrol). They have only one or two phenolic rings.

Alternatively, polyphenols can be divided into four different classes: (1) those containing the pyrocatechol unit (1,2 benzene diol) (hydroxytyrosol, caffeic acid, curcumin); (2) those containing the resorcinol unit (1,3 benzene diol) (resveratrol, genistein); (3) those containing both the pyrocathecol and resorcinol units (quercetin, catechins, anthocyanidins); (4) those containing the pyrogallol unit (1,2,3 benzene triol) (Epigallocathechin) (Figure 6). This classification might are useful because different chemical groups might have different effects. For example, it has been reported that catechols have antiinflammatory activity in stimulated microglia and neutrophils and thereby be useful for the treatment of neurodegenerative diseases [56, 57].

The most important polyphenols are quercetin (QRC), resveratrol (RSV), curcumin (CRC); hydroxytyrosol, catechins, daidzein, genistein. Among the carotenoids the most important is lycopene. They often have a complementary activity as antioxidants and radical scavengers. 

Quercetin is found in onion, apple, citrus, and wine [58]. QRC occurs mainly as glycoside, with a sugar group bound to one of the hydroxyl groups of the flavonol. QRC has antiinflammatory, immunomodulating and antiviral properties, reduces the proliferation of peripheral blood mononuclear cells (PBMCs) and decreases the production of IL-1*β*, TNF-*α* and MMP-9. These effects are additive to those of IFN-*β* [59]. 

QRC passes the BBB [60], inhibits myelin phagocytosis by blocking the ROS released from the macrophages [61] and inhibits the expression of inflammatory cytokines through inhibition of NF-*κ*B [62]. Furthermore, QRC inhibits angiogenesis [63], reduces the neutrophil dependent inflammation [57], and has neuroprotective effects [64]. QRC inhibits or ameliorates the Experimental Allergic Encephalomyelitis (EAE) induced in SJL/J mice by mouse spinal cord homogenate [65]. Apparently, QRC is not toxic [66], but its oxidation product, quercetin quinone (QQ), which is very reactive towards protein thiols and glutathione, could have a toxic effect [67].

Resveratrol is found in red wine, chocolate, peanuts, berries, and black grapes. RSV is glucuronated in the liver and absorbed in this form mainly in the duodenum. Metabolic modification of RSV is inhibited by QRC. Only a few unmodified RSV molecules are absorbed. RSV has a neuroprotective effect and ameliorates MOG-EAE in C57BL/6 mice [68].

RSV has anticarcinogenic, antiinflammatory, and estrogenic activities and allows cardiovascular protection, free-radical scavenging, inhibition/induction of apoptosis, and inhibition of platelet aggregation. Depending on its concentration, RSV mediates cell death of a variety of cells by necrosis or apoptosis downregulating targets such as NF-*κ*B, AP-1, cyclins, STAT 3, and others, and upregulating cytochrome c, caspases, cathepsin D, p53, JNK, and others. RSV acts as a nonsteroidal antiinflammatory molecule and inhibits the production of TNF-*α*, IL-1*α*, IL-1*β*, IL-6, IL-8, IL-18, MMP-9, VEGF, COX-2, 5-lipoxygenase, and ROS. [69, 70].

Moreover, RSV activates the sirtuins, a family of histone deacetylases, particularly SIRT2, and PPAR-*α*/*γ* [71].

Curcumin (CRC) is the yellow pigment present in curry powder. Among its many biological properties, the most important in the present context are its antiinflammatory properties and in particular the downregulation of NF-*κ*B and AP-1 [72]. 

Catechins are polyphenols found in green tea. They have antiinflammatory and anticarcinogenic activity [73], inhibit the activity of MMP-2, MMP-9 and MMP-12 [74] and the intestinal absorption of lipids [75].

Lycopene is a carotenoid that is found in tomato, water-melon, and pink grapefruit. As an antioxidant, it is two times better than beta-carotene and 100 times better than vitamin E and protects against cancer [76, 77]. 

Hydroxytyrosol is the main antioxidant found in olive oil and a very efficient scavenger of free radicals [78]. 

Soy flavonoids as genistein downregulate proimatory cytokines and ameliorate MOG-EAE symptoms in C57BL/6 mice [79].

Thiol-containing compounds such as alpha-lipoic acid (ALA), glutathione and N-acetylcysteine (NAC) might be effective as oral supplements in the complementary treatment of MS. ALA has immunomodulating effects, stimulates the production of cAMP, inhibits the synthesis of IFN-gamma and adhesion molecules [80], is effective in the treatment of EAE [81], affects T cell migration into CNS and stabilizes blood-brain barrier integrity [82]. Studies in patients with multiple sclerosis with oral supplements of lipoic acid are underway to assess the right dosage of this thiol compound [83]. NAC pass through the BBB and, like glucorticoids and interferons, is able to protect it against inflammation [84]. ALA, NAC, and glutathione could be useful to reduce the possible toxic effect of quercetin quinones described above. Other useful compounds with antioxidant activity and a possible role as dietary supplements are: melatonin, vitamin. E, selenium, vitamin C. 

Vitamin D is another candidate for the environmental effects in MS etiology [85–87]. Indeed, the geographic distribution of MS parallels that of the regions with minor exposure to sunlight and lower availability of vitamin D in the population. Accordingly, patients with MS are often deficient in vitamin D. At present, vitamin D represent, the most promising natural molecule for the treatment of autoimmune diseases and MS. Direct gene-environment interaction of vitamin D and its immunomodulatory role in the nervous system may represent the key for disease prevention, but its role is complex and it is not yet sufficiently clear how it exerts a protective effect. 

Vitamin B12 is useful in EAE [88]. Selenium, a constituent of selenoproteins, is considered a dietary antioxidant that could be useful in the treatment of chronic diseases, but its supplementation on long-term basis in healthy individuals is strongly questioned. It is well absorbed as selenomethionin or selenocysteine [89].

### 5.2. Unsaturated Fatty Acids from Vegetables, Sea Food and Fish Oil

Unsaturated vegetable oils are the alternative to saturated animal fat. Olive oil, in particular, should be preferred to seed oils for its optimal ratio between saturated and unsaturated fat. Olive oil contains the antioxidant hydroxytyrosol and the omega-9 (n-9) monounsaturated oleic acid. Vegetable oils contain also the essential fatty acids (EFA) linoleic acid (n-6) and linolenic acid (n-3). n-6 and n-3 fatty acids have opposing effects and their intake in the diet should be equivalent [90]. However, in Western diets the n-6/n-3 ratio is increased 6/15-fold- and this causes a greater incidence of cardiovascular and inflammatory diseases. Indeed, the n-6 fatty acid linoleic acid leads to the production of the 20 : 4 arachidonic acid (AA), which is the precursor of proinflammatory eicosanoids such as prostaglandins (2-series), leukotriens (4-series), and tromboxanes (2-series). Synthesis of these eicosanoids is favored by insulin and inhibited by aspirin, but also by the n-3 long chain polyunsaturated fatty acids (PUFAs), that is, eicosapentaenoic acid (EPA) and docosahexaenoic acid (DHA), produced from the n-3 linolenic acid.

DHA is present at high concentration in the brain but its levels decrease dramatically in MS patients. Both EPA and DHA are found in high proportion in oily fish and fish oil and show remarkable antiinflammatory, antithrombotic and immunomodulating activities which are comparable with statins. They also exert a number of neuroprotective effects and have a therapeutic value in several neurological diseases [91–93].

n-3 PUFAs exert important effects on gene expression. They inhibit the transcription factors NF-*κ*B, LXR and SREBP-1c (the sterol regulatory element binding protein 1c) and activate the nuclear receptors PPAR. As a result, n-3 PUFA decrease inflammatory processes and the synthesis of fatty acids, but increase fatty acid oxidation [90]. On these grounds, dietary n-3 EFA should prevail over n-6 EFA in inflammatory diseases such as MS.

In LPS-activated rat microglial cells, fish oil inhibits the expression of MMP-9, an important mediator of neuroinflammation involved in myelin breakdown [94], in a similar way as IFN-beta does [95]. Like IFN-beta, EPA and DHA also inhibit the formation of IFN-gamma. DHA also increases the levels of tissue inhibitor of metalloproteinase-1 (TIMP-1) [96].

Moreover, n-3 PUFA significantly decreased MMP-9 levels in relapsing-remitting MS (RR-MS) [97], and other few clinical trials indicate that n-3 PUFA may represent a good complementary treatment in the course of MS [98, 99]. Fish oil has been also found to improve motor performances in healthy rat pups [100].

Seeds oils, from sunflower, corn, soy bean, and sesame, contain more n-6 fatty acids (FAs) than n-3 FAs, and their assumption should be limited in MS, in order to limit the extent of proinflammatory eicosanoid production. Coconut oil has a high content of saturated FAs. It is useful to know that the intake of PUFA should be integrated with antioxidants. 

Hydrogenated FAs, found in margarine and other treated fat, are *trans* FAs and not *cis* FAs, like the natural ones, and interfere with PUFA metabolism. Similarly, the frequent intake of fried should be avoided, as fry causes the conversion of *cis* FA in *trans* FAs.

### 5.3. Multiple Dietary Supplements

As indicated above, bioactive natural products have a pleiotropic nature and act on several and different targets in the cell. Moreover, antioxidants have different redox potentials and use different mechanisms as radical scavengers. On these grounds, it can be expected that an appropriate combination of some of them may allow a synergistic effect at low concentrations and this may decrease the risk of toxicity [101]. For example, polyphenols act mainly as antioxidants but their oxidation may produce oxygen radicals and quinones which are toxic [102]. 

A cocktail of polyphenols differing in antioxidant capacity and in the action on cellular targets can cover a large spectrum of activities, in particular in combination with n-3 PUFA. The utility of multiple dietary supplements administration has been shown in recent experimental works [70, 103–105].

### 5.4. Calorie Restriction (CR)

Diet does not mean only the intake of bioactive dietary compounds. The most prominent aspect that can augment the risk of chronic diseases is energy intake: excessive calorie intake increases the risk. Calorie restriction, obtained either by decrease in food intake or intermittent fasting (“every-other-day fasting”), protects against neurodegenerative disease models and might be effective in slowing the progression of MS [106, 107]. CR decreases the extent of oxidative damage and its effects can be mimicked by RSV.

### 5.5. The Role of Insuline in MS

Excessive calorie intake increases the production of free radicals and inflammation. Moreover, a meal rich in carbohydrates increases insulin levels which stimulates lipogenesis and activates the enzyme 5-delta-desaturase (inhibited by EPA) which forms the n-6 PUFA arachidonic acid (AA) from dihomo-gamma-linoleic acid (DGLA). AA forms the eicosanoids involved in inflammation.

### 5.6. Physical Exercise

In general, endurance exercise exerts beneficial effects on health by targeting the AMP-activated protein kinase AMPK and the AMPK-PPAR delta pathway. AMPK plays a key role in energy balance in the cell and leads to suppression of ATP-consuming anabolic pathways as lipogenesis and induction of ATP forming catabolic pathways as fatty acid oxidation. AMPK is a target of adipokines (leptin and adiponectin) and regulate food intake, body weight, as well as glucose and lipid homeostasis. RSV and AMPK agonists can mimic and enhance the effects of training [108]. Exercise training induces a decrease in plasma leptin levels and a reduction in gene expression of leptin receptors in liver [109]. The AMPK system appears to be downregulated in EAE, and AMPK agonists and metformin, a drug used to treat diabetes, attenuate the disease [110, 111].

## 6. Discussion

MS is a complex, multifactorial disease and nutrition is an environmental factor possibly involved in its pathogenesis. However, the role of nutrition in MS is at present completely disregarded both in treatment and study of MS, and MS therapy is not associated to a particular diet. 

In this article we have tried to answer to some fundamental questions regarding the relationship between nutrition and MS.

The first question is whether nutritional habits really might have something to do with MS. The 2008 WHO report clearly indicates that MS has a geographical distribution corresponding to countries with hypercaloric, high-fat/high-carbohydrate diets and reduced exposure to ultraviolet sunlight. Both diet and sunlight exposure may in fact explain the influence of migration in young age on the course of the disease and the lower availability of vitamin D in MS patients. 

Dietary habits are not relevant *per se* but, as shown in Figure 2, hypercaloric diets promote the synthesis of fat and inflammatory molecules. Accordingly, a study of the North American Research Committee on Multiple Sclerosis has recently found that a quarter of the patients were obese, and 31.3% were overweight [112]. On these grounds, a low calorie (1700 kcal/day), low-fat diet, based on the consumption of vegetables, fruit and fish, if possible varied (multicolored) and distributed five times the day, should represent in the future an important complementary treatment in the course of MS in addition to pharmacological therapy. 

The second question we tried to answer in this paper was to assess at molecular level how diet and dietary supplements may ameliorate the course of MS and improve the wellness of MS patients. As shown here, calorie restriction, physical exercise, dietary antioxidants from vegetables and fruits, as well as polyunsaturated fatty acids from fish, are able to counteract the inflammatory responses associated to MS by downregulating nuclear receptors, transcription factors, and enzymes involved in the proimatory processes. For this reason, the interest in natural bioactive compounds, mainly belonging to the class of polyphenols and to n-3 PUFA, as coadjuvants in the treatment of chronic diseases is increasing very rapidly. 

In particular, recent data are showing that polyphenols (i) downregulate the expression of a number of enzymes and molecules (5-LOX, COX-2, iNOS, MMP-9, IL-8, IL-6, TNF-*α*, IL-12 and adhesion molecules such VCAM-1 and ICAM-1); (ii) upregulate the PPARs, in particular *α* and *γ*, and Nrf2; downregulate NF-*κ*B, AP-1, STAT-1,3,5; (iii) inhibit several protein kinases (IKK, EGFR, HER-2, AKT, JAK-2, Src, JNK, PKA, PKC, TYK-2 ...) and enzymes such as GST, GSH-px, uPA, FTPase, xanthine oxidase, hemeoxygenase. In most cases polyphenols and n-3 PUFA share common effects. All these data indicate that natural bioactive molecules might be able to influence the course of MS and ameliorate the wellness of MS patients through molecular mechanisms that appear plausible and convincing. Molecular targets and mechanisms of action of natural and pharmacological drugs are often the same. This means that we can expect that they can act in synergy. 

We have recently shown that flavonoids and nonflavonoids inhibit MMP-9 levels with different mechanisms possibly related to their different structure: flavonoids, such as quercetin and the cathechins from green tea, inhibit MMP-9 directly; while non flavonoids, such as resveratrol and tyrosol/hydroxytyrosol from olive oil, inhibit its expression [113].

Based on the investigations carried out in cancer and rheumatoid arthritis, polyphenols seem to have the potential for their treatment, as well as of various chronic inflammatory diseases, but very few clinical trials have yet been done with the pure compounds. At present, only the studies on EAE can give some indication on the uselfulness of the treatment with antioxidants in MS. With the exception of the study of Verbeek et al. [114], all antioxidants tested appear to exert a healthy effect on the disease.

Data on the bioavailability of polyphenols in humans are not yet sufficient and bioavailability of polyphenols is, in any case, very low. In foods, flavonoids are mainly glycosylated and phenolic acids are in the form of esters and, after their conversion to aglycons, they are sulphated, glucuronidated or methylated in our body [115, 116]. This makes difficult to determine the optimal dose of polyphenols required to achieve protective effects and avoid the formation of toxic compounds.

Moreover, it is not clear whether these bioactive molecules should be taken alone or in combination and which is their optimal concentration or the best ratio. The recommended daily allowances (RDAs) of polyphenols are not yet known and it is necessary to assess the lowest concentration, which is effective in counteracting cell activation without the formation of high amounts of toxic oxidation products. An excess of antioxidants can indeed be deleterious. Protection from quinones and other toxic compounds can be achieved by –SH containing products such as alfa-lipoic acid and N-acetylcysteine.

At present, polyphenols are taken in the order of mg and PUFA in the order of grams, but decisions concerning the dosage of dietary supplements and their combination should be taken in studies *in vitro *and *in vivo *experiments. In particular, clinical studies with polyphenols, n-3 PUFA and vitamin D and other antioxidants should be taken into consideration for their effectiveness in MS patients. 

In conclusion, given that diet and dietary supplements are not pharmacological drugs and cannot replace the conventional MS therapy, they can concur to the amelioration of the wellness of MS patients. As shown in this review paper, the fundamental molecular prospect for a nutritional intervention in inflammatory, autoimmune diseases as MS, is given by the ability of some natural food components to modulate cell metabolism and in particular the activity of ligand-dependent nuclear receptors and transcription factors, which are able to downregulate inflammatory and autoimmune processes. Among them, the most promising targets of dietary molecules are the PPAR, in particular PPAR-gamma, that have a pivotal role in regulating both metabolism and inflammation [117]. Future research will clarify whether bioactive dietary molecules could also influence the process of differentiation of mature oligodendrocytes and promote remyelination.
